# Generation of 500-Member Library of 10-Alkyl-2-R^1^,3-R^2^-4,10-DihydrobenzoDihydrobenzo[4,5]imidazo[1,2-*a*]pyrimidin-4-ones

**DOI:** 10.3390/molecules14125223

**Published:** 2009-12-15

**Authors:** Svetlana M. Sirko, Nikolay Yu. Gorobets, Vladimir I. Musatov, Sergey M. Desenko

**Affiliations:** Department of Chemistry of Heterocyclic Compounds, SSI “Institute for Single Crystals” of National Academy of Sciences of Ukraine, Lenin Ave. 60, Kharkiv 61001,Ukraine

**Keywords:** benzimidazole, Dihydrobenzo[4,5]imidazo[1,2-*a*]pyrimidin-4-one library, regioselective alkylation, anti-cancer agents

## Abstract

Representative benzimidazopyrimidinones were previously reported to be intercalating antitumor agents. In this work, we used 2-substituted 4,10-dihydrobenzo Dihydrobenzo[4,5]imidazo[1,2-*a*]pyriminin-4-ones for their diversification by regioselective alkylation. Under the conditions established, the alkylation gave 10-alkyl derivatives which permitted the parallel generation of a 500-member library of the title compounds.

## Introduction

The benzimidazole fragment is a frequent motif in recent publications devoted to drug design and molecular diversity oriented synthesis [1,2,3,4,5,6,7,8,9]. Fused polycyclic derivatives of benzimidazo[1,2-*a*] pyrimidines are often cited as anti-cancer and cytotoxic agents [10,11,12,13,14]. These molecules bind to DNA by stacking interactions of their π-electron rich planar fragments with the nucleic acid sandwiching in two consecutive base pairs of the double helix. This affects the DNA shape eventually prohibiting replication and causing the cell death [15,16].

**Figure 1:**
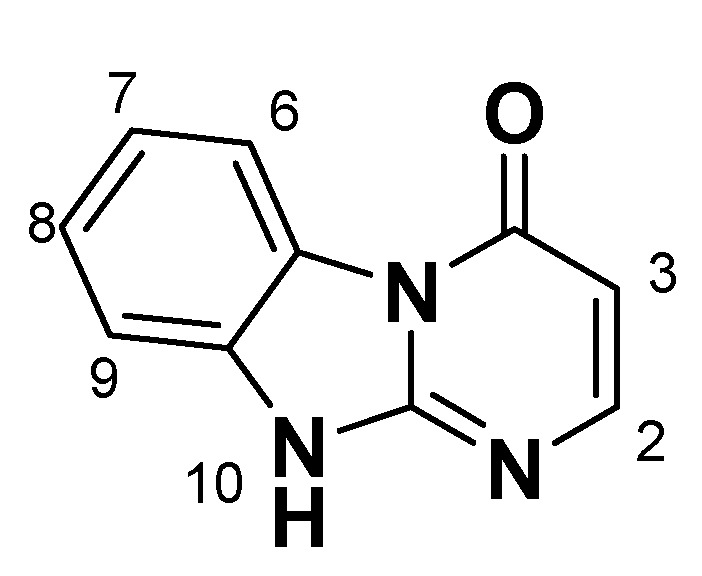
Dihydrobenzo[4,5]imidazo[1,2-*a*]pyrimidin-4-one core.

Condensed benzimidazoles represent a suitable platform for the construction of such planar molecules applying the strategy of diversity oriented synthesis. We were interested in the generation of a library of diverse compounds based on the Dihydrobenzo[4,5]imidazo[1,2-*a*]pyrimidin-4-one core (Figure 1).

We aimed to generate a 500-member library of Dihydrobenzo[4,5]imidazo[1,2-*a*]pyrimidin-4-ones by introducing the initial diversity points in the positions C-2 and C-3 and then diversifying the obtained compounds by regioselective alkylation reactions using alkylating agents of different types in parallel format. In view of the scaffold structure (Figure 1) there are at least three products of monoalkylation that can be expected: the N1- and N10-substituted derivatives and the product of O-alkylation. Thus, uniform conditions for regioselective alkylation of Dihydrobenzo[4,5]imidazo[1,2-*a*]pyrimidin-4-ones are required.

## Results and Discussion

Several synthetic approaches to the Dihydrobenzo[4,5]imidazo[1,2-*a*]pyrimidin-4-one scaffold **1** have been found in the literature [17,18,19,20,21,22]. All of them utilize the reactivity of 2-aminobenzimidazole **2** with 1,3-dielectrophiles of several types. However, because of the commercial ability of different β-ketoesters **3**, we have applied them [23] for the synthesis of starting compounds **1a–p**. In contrast to the original protocol [23] where authors heated a neat mixture of 2-aminobenzimidazole **2** and some liquid β-ketoesters **3**, we applied DMF as a solvent to make the procedure general for different starting materials including solid esters **3e–h **(Table 1). Application of other solvents (EtOH, AcOH, dioxane) at reflux resulted in lower yields.

**Scheme 1:**
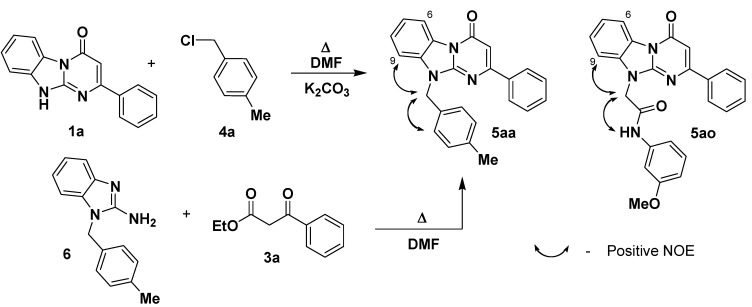
Model alkylation of the 2-phenyl-Dihydrobenzo[4,5]imidazo[1,2-*a*]pyrimidin-4-one **1a**.

In our initial experiments on the alkylation we used the representative **1a** in the reaction with 4-methylbenzyl chloride **4a** under different reaction conditions. Strong alkali media (NaH or KOH in DMF, DMSO or dioxane) gave mixtures of alkylation products and therefore were not acceptable. Milder reaction conditions (NaHCO_3_ or K_2_CO_3_ in acetone, NEt_3_ in DMF) led to low yields or to products contaminated with the starting material **4a**. However, application of K_2_CO_3_ (3 equiv) in DMF at 90 °C for 2h resulted in the formation of only one isomer **5aa** with 81% yield following a simple aqueous workup (Scheme 1). Application of such conditions is very convenient for parallel synthesis because the reaction media does not reflux and the reaction can be carried out in a simple sealed vessel. These conditions were suitable for application of a representative of N-substituted 2-chloroacetamides **4o** (Table 2). The product **5ao** was obtained in the same manner with 77% yield (Scheme 1).

**Table 1:** Synthesis of exampled starting Dihydrobenzo[4,5]imidazo[1,2-*a*]pyrimidin-4-ones **1a–p**.

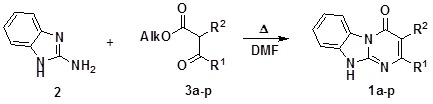					
Code	Structure	Yield,%	Code	Structure	Yield,%
**1a**	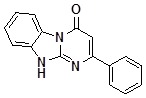	75	**1i**	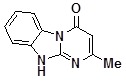	60
**1b**	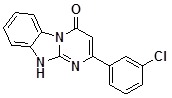	80	**1j**	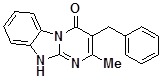	47
**1c**	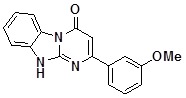	71	**1k**	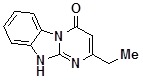	54
**1d**	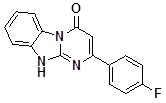	79	**1l**	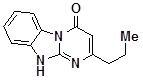	56
**1e**	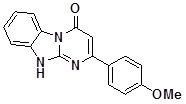	87	**1m**	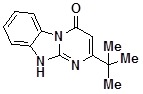	73
**1f**	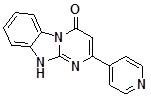	87	**1n**	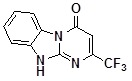	55
**1g**	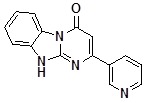	77	**1o**	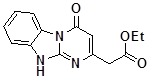	35
**1h**	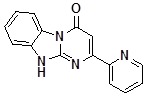	80	**1p**	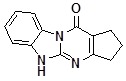	67

**Table 2:** Selected examples **4a–u** of alkylation agents used for the library **5** generation.

**Code**	Structure	**Code**	Structure	**Code**	Structure
**4a**	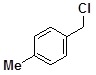	**4h**	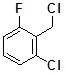	**4o**	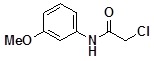
**4b**	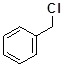	**4i**	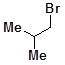	**4p**	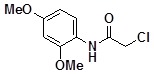
**4c**	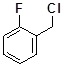	**4j**	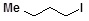	**4q**	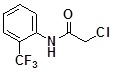
**4d**	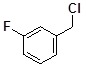	**4k**	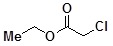	**4r**	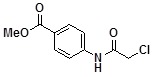
**4e**	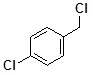	**4l**	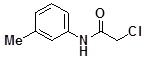	**4s**	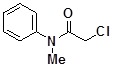
**4f**	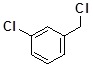	**4m**	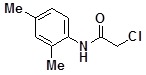	**4t**	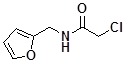
**4g**	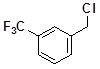	**4n**	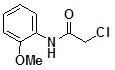	**4u**	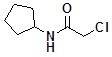

The information on alkylation of the Dihydrobenzo[4,5]imidazo[1,2-*a*]pyrimidin-4-ones **1** turned out to be absent in the literature, thus it was not possible to predict with certainty which isomer out of three mentioned above was formed. The NOE experiment, irradiation of the product **5aa** with the resonance frequency of its CH_2_ protons at 5.58 ppm has demonstrated a close location of the methylene group and the upfield CH proton of the benzimidazole ring (doublet at 7.63 ppm) which agrees with either for O-alkylated or N10-alkylated product. To undoubtedly determine the structure of the product **5aa** we have carried out a counter-synthesis (Scheme 1) of this compound starting from 2-amino-1-(4-methylbenzyl)benzimidazole **6** described previously [24]. The products obtained in these two alternative ways were identical. This means that the upfield doublet (at 7.63 ppm) of the benzimidazole ring belongs to the 9-C-H proton but not to the 6-C-H. Consequently, the latter gives its downfield doublet at 8.48 ppm. This alternative synthetic pathway leading to the compound **5aa** gives lower yield (54% isolated yield). Also in this protocol the main diversity point (the alkylation agent) was introduced not at the last synthetic step, which was less convenient for the library generation. Alkylation of the starting derivative **1a** using a representative of the N-substituted 2-chloroacetamides **4o** led to one regioisomer **5ao**. The discussed signal assignment for compound **5aa** gives opportunity to determine structure of the product **5ao**, the positive NOE between the protons of the CH_2_ group (5.29 ppm) and the upfield benzimidazole 9-C-H proton (doublet at 7.71 ppm) fully confirms the N10-alkylation.

The specified conditions allowed us to perform regioselective alkylation of the 2-(het)arylderivatives **1a–g** with all the applied chemotypes of alkylation agents (representatives of benzyl clorides, 2-chloroacetic acid derivatives and representatives of alkylbromides or iodides, see Table 2 for selected alkylation agents **4a–u**). However, a series of pilot experiments with the alkylation of 2-methyl-Dihydrobenzo[4,5]imidazo[1,2-*a*]pyrimidin-4-one **1i** by representatives of the N-substituted 2-chloroacetamides **4l–u** led to isolation of a mixture of two isomeric products. At the same time, the application of other alkylation agents was successful in this case. The same result was observed for the 2-*tert*-butyl derivative **1m**. Also, in the case of the starting compounds **1o**,**p**, we could not achieve the regioselectivity with alkylators of all chemotypes. Thus, we had to exclude the 2-alkyl derivatives **1i–n** from the starting set used in the alkylation with the N-substituted 2-chloroacetamides **4l–u**, and we did not use the derivatives **1o**,**p** for the library generation.

**Scheme 2:**
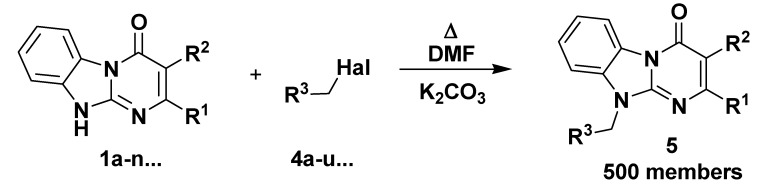
Generation of a 500-member library of 10-alkyl-2-R^1^,3-R^2^-4,10-Dihydrobenzo[4,5]imidazo[1,2-*a*]pyrimidin-4-ones **5**.

**Figure 2:**
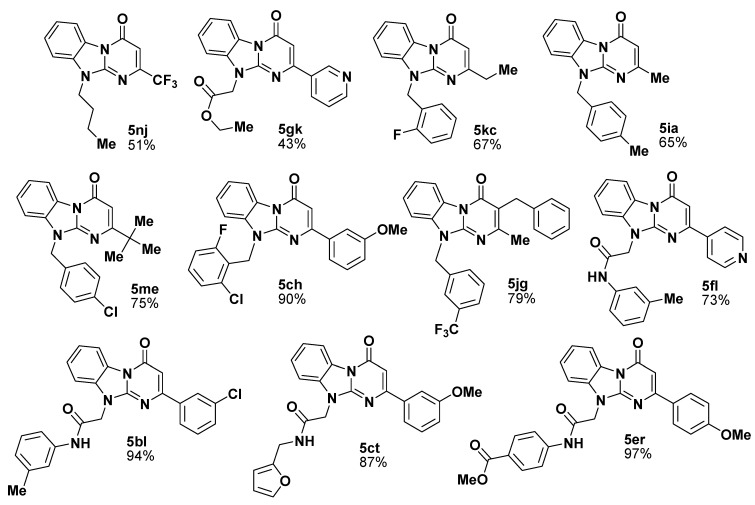
Selected members of the generated library **5** and their isolated yields.

**Figure 3:**
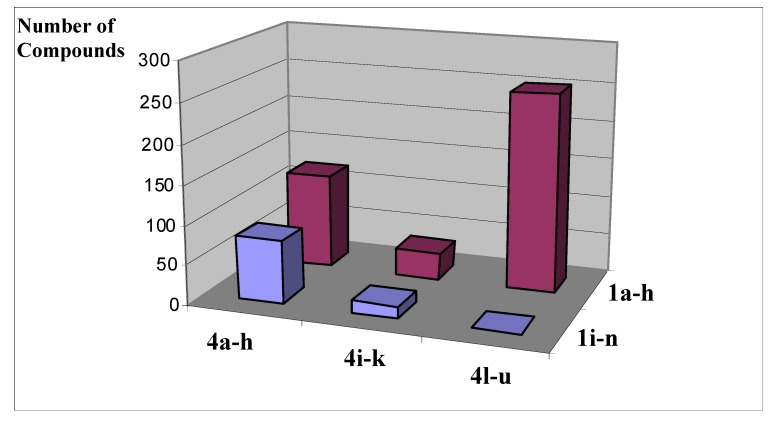
Distribution of different congener’s chemotypes in the generated 500-member library **5**.

The library was generated in parallel format (Scheme 2, Figure 2) and the structure and purity of every congener were checked by ^1^H-NMR. In about 85% cases the products were isolated with very good average yield (about 70%) and met the purity requirements (maximal total level of impurities less then 10% as determined by ^1^H-NMR). In those cases (about 10%) where the impurity level was higher than 10% the product purity was improved by heating in EtOH to remove soluble remains of the starting materials. In about 5% of cases the products did not satisfy the purity requirements either after the simple workup or after the additional purification.

As a result, the desired 500-member library contained different compound chemotypes whose distribution is illustrated in Figure 3. As one can see, the starting 2-(het)arylderivatives **1a–g** contributed much more significantly to the total number of compounds because of their selectivity in the reaction with the diverse N-substituted 2-chloroacetamides **4l–u**.

In summary, a 500-member library of 10-Alkyl-2-R^1^,3-R^2^-4,10-Dihydrobenzo[4,5]imidazo[1,2-*a*]pyrimidin-4-ones was quickly generated under uniform conditions in liquid phase parallel format. Structural diversity of the library members was achieved by application of two rows of starting Dihydrobenzo[4,5]imidazo[1,2-*a*]pyrimidin-4-ones containing different 2-(het)aryl (**1a–h**) or 2-alkyl (**1i–n**) substituents as well as three types of alkylation agents. The major part of congeners comprises conjugated planar molecules derived from 2-(het)arylDihydrobenzo[4,5]imidazo[1,2-*a*]pyrimidin-4-ones **1a–h** that makes them potential anti-cancer DNA intercalators.

## Experimental

### General

The starting Dihydrobenzo[4,5]imidazo[1,2-*a*]pyrimidin-4-ones **1a** [25], **1e** [26], **1i** [25], **1o [27]**, **1p** [27] have been previously described. The starting sets of ß-ketoesters and alkylation agents were supplied by Chemical Diversity Research Institute, Chimki, Moscow, Russia. All other reagents and solvents are commercially available and were used without additional purification. ^1^H, ^13^C and NOE NMR experiments were recorded at 200 MHz (50 MHz for ^13^C NMR) in DMSO-d_6_ solutions.

*Dihydrobenzo[4*,*5]imidazo[1*,*2-a]pyrimidin-4-ones* (**1a–p**). An equimolar mixture of 2-amino-benzimidazole **2** and corresponding β-ketoester **3a–p ** (0.04 mol) in DMF (4 mL) was refluxed for 20–60 min. If a precipitate was formed or the mixture was solidified at heating, the mixture was kept at mild heating for another 5 min and then cooled down to the room temperature. In the cases when no precipitate was formed, the mixture was refluxed for 60 min and then cooled down to the room temperature. The solid reaction mixture was fluidized with EtOH, the precipitated was separated by filtration, washed with EtOH and dried on air. The products **1a-p** obtained in this manner had adequate ^1^H NMR spectra and used for the next reaction step without additional purification.

*2-(3-Chlorophenyl)dihydrobenzo[4*,*5]imidazo[1,2-a]pyrimidin-4-one* (**1b**). ^1^H-NMR δ 6.70 (s, 1H), 7.31 (m, 1H), 7.41-7.62(m, 4H), 8.05(m, 1H), 8.14 (m, 1H), 8.44 (dd, *J* = 1.2 Hz, *J* = 7.9 Hz, 1H), 13.12 (br. s, 1H).

*2-(3-Methoxyphenyl)dihydrobenzo[4*,*5]imidazo[1,2-a]pyrimidin-4-one* (**1c**). ^1^H-NMR δ 3.83 (s, 3H), 6.64 (s, 1H), 7.06 (m, 1H), 7.20-7.57 (m, 4H), 7.57-7.83 (m, 2H), 8.46 (d, *J* = 7.9 Hz, 1H), 13.06 (br. s, 1H).

*2-(4-Fluorophenyl)dihydrobenzoDihydrobenzo[4,5]imidazo[1,2-a]pyrimidin-4-one* (**1d**). ^1^H-NMR δ 6.61 (s, 1H), 7.22-7.42 (m, 3H), 7.47 (d, *J* = 4.0 Hz, 2H), 8.16 (m, 2H), 8.45 (dd, *J* = 0.7 Hz, *J* = 7.8 Hz, 1H), 13.10 (br. s, 1H).

*2-(4-Metoxyphenyl)dihydrobenzoDihydrobenzo[4,5]imidazo[1,2-a]pyrimidin-4-one* (**1e**). ^1^H-NMR δ 3.82 (s, 3H), 6.50 (s, 1H), 7.05 (d, *J* = 8.7 Hz, 2H), 7.17-7.60 (m, 3H), 8.07 (d, *J* = 8.7 Hz, 2H), 8.44 (d, *J* = 7.9 Hz, 1H), 12.95 (br. s, 1H); ^13^C NMR δ 56.1, 96.6, 111.8, 114.8, 116.3, 122.4, 126.6, 126.8, 129.3, 130.2, 131.8, 150.3, 160.4, 161.1, 162.0.

*2-(Pyridin-4-yl)dihydrobenzoDihydrobenzo[4,5]imidazo[1,2-a]pyrimidin-4-one* (**1f**). ^1^H-NMR δ 6.80 (s, 1H), 7.36 (m, 1H), 7.50 (d, *J* = 4.0 Hz, 2H), 8.04 (dd, *J* = 1.7 Hz, *J* = 5.2 Hz, 2H), 8.47 (d, *J* = 7.9 Hz, 1H), 8.71 (dd, *J* = 1.7 Hz, *J* = 5.2 Hz, 2H), 13.10 (br. s, 1H).

*2-(Pyridin-3-yl)dihydrobenzoDihydrobenzo[4,5]imidazo[1,2-a]pyrimidin-4-one* (**1g**). ^1^H-NMR δ 6.73 (s, 1H), 7.33 (m, 1H), 7.42-7.60 (m, 3H), 8.33-8.56 (m, 2H), 8.66 (dd, *J* = 1.7 Hz, *J* = 4.7 Hz, 1H), 9.27 (dd, *J* = 0.8 Hz, *J* = 2.3 Hz, 1H).

*2-(Pyridin-2-yl)dihydrobenzoDihydrobenzo[4,5]imidazo[1,2-a]pyrimidin-4-one* (**1h**). ^1^H-NMR δ 7.03 (s, 1H), 7.35 (m, 1H), 7.41-7.63 (m, 3H), 7.98 (td, *J* = 0.8 Hz, *J* = 7.9 Hz, 1H), 8.32 (d, *J* = 7.9 Hz, 1H), 8.47 (d, *J* = 7.9 Hz, 1H), 8.70 (d, *J* = 0.5 Hz, 1H), 13.17 (br. s, 1H).

*3-Benzyl-2-methyldihydrobenzoDihydrobenzo[4,5]imidazo[1,2-a]pyrimidin-4-one* (**1j**). ^1^H-NMR δ 2.30 (s, 3H), 3.88 (s, 2H), 7.03-7.35 (m, 6H), 7.35-7.60 (m, 2H) 8.39 (d, *J* = 7.8 Hz, 1H), 12.32 (br. s, 1H).

*2-EthyldihydrobenzoDihydrobenzo[4,5]imidazo[1,2-a]pyrimidin-4-one* (**1k**). ^1^H-NMR δ 1.20 (t, *J* = 7.5 Hz, 3H), 2.56 (q,*J* = 7.5 Hz, 2H), 5.83 (s, 1H), 7.26 (td, *J* = 1.5 Hz, *J* = 7.9 Hz, 1H), 7.34-7.59 (m, 2H), 8.35 (d, 7.8 Hz, 1H), 12.66 (br. s, 1H).

*2-PropyldihydrobenzoDihydrobenzo[4,5]imidazo[1,2-a]pyrimidin-4-one* (**1l**). ^1^H-NMR δ 0.90 (t, *J* = 7.2 Hz, 3H), 1.67 (hexet, *J* = 7.2 Hz, 2H), 2.42-2.55 (m, 2H), 5.82 (s, 1H), 7.25 (td, *J* = 1.5 Hz, *J* = 7.9 Hz, 1H), 7.33-7.60 (m, 2H), 8.35 (d, 7.9 Hz, 1H), 12.67 (br. s, 1H);^ 13^C-NMR δ 14.0, 21.7, 38.1, 98.9, 113.8, 115.9, 122.0, 126.2, 127.5, 135.2, 149.4, 160.0, 164.1.

*2-tert-ButyldihydrobenzoDihydrobenzo[4,5]imidazo[1,2-a]pyrimidin-4-one* (**1m**). ^1^H-NMR δ 1.27 (s, 9H), 5.95 (s, 1H), 7.27 (m, 1H), 7.33-7.53 (m, 2H), 8.37 (d, 7.9 Hz, 1H), 12.85 (br. s, 1H).

*2-TrifluoromethtyldihydrobenzoDihydrobenzo[4,5]imidazo[1,2-a]pyrimidin-4-one* (**1n**). ^1^H-NMR δ 6.43 (s, 1H), 7.36 (m, 1H), 7.47-7.58 (m, 2H), 8.44 (dd, *J* = 0.8 Hz, *J* = 7.9 Hz, 1H).

### Generation of 500-member Library ***5*** of 10-Alkyl-2-R^1^,3-R^2^-4,10-Dihydrobenzo[4,5]imidazo[1,2-a]pyrimidin-4-ones

The equimolar mixture (0.6 mmol) of starting Dihydrobenzo[4,5]imidazo[1,2-*a*]pyrimidin-4-one (**1a–n**...) and corresponding alkylation agent (**4a–u**…) was dissolved in DMF (2 mL) with mild heating. Then K_2_CO_3_ (1.8 mmol) was added and the reaction mixture was stirred at 90-100 °C for 2h. The mixture was cooled down and diluted with 50% aqueous EtOH (4 mL). The formed precipitate was removed by filtration, washed with H_2_O and 50% aqueous EtOH and dried on air at 90 °C. In those cases where ^1^H-NMR demonstrated more than 10 mol.% total impurity level, the product was additionally purified by heating in 1-2 mL of EtOH for 3-5 min, then cooling to rt, and isolation by filtration.

*10-(4-Methylbenzyl)-2-phenyldihydrobenzoDihydrobenzo[4,5]imidazo[1,2-a]pyrimidin-4-one* (**5aa**). ^1^H-NMR δ 2.22 (s, 3H, Me), 5.58 (s, 2H, CH_2_), 6.72 (s, 1H, 3-CH), 7.13 (d, *J* = 8.1 Hz, 2H, Ar), 7.29-7.43 (m, 3H, Ar), 7.43-7.58 (m, 4H, Ar), 7.63 (d, *J* = 7.9 Hz, 1H, 9-CH), 8.07-8.33 (m, 2H, 7-CH and 8-CH), 8.48 (d, *J* = 7.9 Hz, 1H, 6-CH); ^13^C-NMR δ 21.2, 45.9, 98.4, 110.8, 116.4, 123.1, 125.9, 126.8, 127.8, 128.4, 129.3, 129.9, 131.0, 131.7, 133.5, 137.7, 137.9, 149.6, 160.4, 161.2.

*2-(4-Oxo-2-phenylpyrimido[1,2-a]benzimidazol-10(4H)-yl)-N-(3-methoxyphenyl)acetamide* (**5ao**).^1^H-NMR δ 3.68 (s, 3H, OMe), 5.29 (s, 2H, CH_2_), 6.65 (dd, *J* = 1.7 Hz, *J* = 7.9 Hz, 1H, Ar), 6.73 (s, 1H, 3-CH), 6.98-7.34 (m, 3H, Ar), 7.34-7.62 (m, 5H, Ar), 7.71 (d, *J* = 8.1 Hz, 1H, 9-CH), 8.00-8.27 (m, 2H, 7-CH and 8-CH), 8.51 (d, *J* = 8.1 Hz, 1H, 6-CH), 10.52 (s, 1H, NH).

*2-[4-Oxo-2-(3-chlorophenyl)pyrimido[1,2-a]benzimidazol-10(4H)-yl]-N-(3-methylphenyl)acetamide*

(**5bl**). ^1^H-NMR δ 2.25 (s, 3H), 5.29 (s, 2H), 6.76 (m, 1H), 6.89 (d, *J* = 7.3 Hz, 1H), 7.18 (t, *J* = 8.2 Hz, 1H), 7.27-7.64 (m, 6H), 7.70 (d, *J* = 7.9 Hz, 1H), 8.10 (d, *J* = 6.4 Hz, 1H), 8.20 (s, 1H), 8.53 (d, *J* = 7.9 Hz, 1H), 10.40 (br. s, 1H); ^13^C-NMR δ 21.6, 45.9, 99.1, 110.8, 116.2, 117.7, 121.0, 123.3, 125.1, 125.7, 126.3, 127.0, 127.5, 129.2, 130.7, 131.0, 131.1, 132.5, 134.4, 138.7, 139.0, 139.7, 149.6, 159.4, 160.2, 165.2.

*10-(2-Chloro-6-fluorobenzyl)-2-(3-methoxyphenyl)dihydrobenzoDihydrobenzo[4,5]imidazo[1,2-a]pyrimidin-4-one*

(**5ch**). ^1^H-NMR δ 3.84 (s, 3H), 5.74 (s, 2H), 6.71 (s, 1H), 7.04 (m, 1H), 7.19-7.78 (m, 9H), 8.50 (d, *J* = 7.9 Hz, 1H).

*N-(Furan-2-ylmethyl)-2-[4-Oxo-2-(3-methoxyphenyl)pyrimido[1,2-a]benzimidazol-10(4H)-yl]-*

*acetamide* (**5ct**). ^1^H-NMR δ 3.83 (s, 3H), 4.30 (d, *J* = 5.5 Hz, 1H), 5.11 (s, 2H), 6.23 (dd, *J* = 0.7 Hz, *J* = 3.3 Hz, 1H), 6.32 (dd, *J* = 1.8 Hz, *J* = 3.3 Hz, 1H), 6.75 (s, 1H), 7.07 (m, 1H), 7.31-7.47 (m, 2H), 7.47-7.59 (m, 2H), 7.59-7.79 (m, 3H), 8.49 (d, *J* = 8.1 Hz, 1H), 8.86 (t, *J* = 5.5 Hz, 1H).

*N-(4-Carbomethoxyphenyl)-2-[4-oxo-2-(4-methoxyphenyl)pyrimido[1,2-a]benzimidazol-10(4H)-yl]-*

*acetamide* (**5er**). ^1^H-NMR δ 3.78 (s, 6H), 5.31 (s, 2H), 6.63 (s, 1H), 6.98 (d, *J* = 8.9 Hz, 2H), 7.37 (t, *J* = 7.3 Hz, 1H), 7.51 (t, *J* = 7.3 Hz, 1H), 7.62-7.79 (m, 3H), 7.89 (d, *J* = 8.9 Hz, 2H), 8.09 (d, *J* = 8.9 Hz, 2H), 8.48 (d, *J* = 7.8 Hz, 1H), 10.70 (br. s, 1H); ^13^C-NMR δ 46.0, 52.4, 56.1, 97.2, 110.7, 114.8, 116.2, 119.8, 123.1, 125.4, 125.9, 126.7, 129.4, 129.8, 130.9, 132.5, 143.8, 149.6, 160.3, 160.9, 162.1, 166.0, 166.5.

*N-(3-Methylphenyl)-2-[4-oxo-2-(pyridin-4-yl)pyrimido[1,2-a]benzimidazol-10(4H)-yl]-acetamide*

(**5fl**). ^1^H-NMR δ 2.23 (s, 3H), 5.31 (s, 2H), 6.88 (d, *J* = 7.3 Hz, 1H), 6.93 (s, 1H), 7.18 (t, *J* = 7.8 Hz, 1H), 7.28-7.51 (m, 3H), 7.57 (t, *J* = 8.2 Hz, 1H), 7.73 (d, *J* = 8.2 Hz, 1H), 8.09 (d, *J* = 6.1 Hz, 2H), 8.52 (d, *J* = 7.8 Hz, 1H), 8.69 (d, *J* = 6.1 Hz, 2H), 10.49 (br. s, 1H).

*Ethyl N-(3-methylphenyl)-2-[4-oxo-2-(pyridin-3-yl)pyrimido[1,2-a]benzimidazol-10(4H)-yl]acetate*

(**5gk**). ^1^H-NMR δ 1.21 (t, *J* = 7.0 Hz, 3H), 4.19 (q, 7.0 Hz, 2H), 5.36 (s, 2H), 6.87 (s, 1H), 7.32-7.66 (m, 3H), 7.74 (d, *J* = 7.9 Hz, 1H), 8.49 (d, *J* = 8.1 Hz, 2H), 8.67 (dt, *J* = 1.7 Hz, *J* = 4.7 Hz, 1H), 9.33 (m, 1H).

*2-Methyl-10-(4-methylbenzyl)dihydrobenzoDihydrobenzo[4,5]imidazo[1,2-a]pyrimidin-4-one* (**5ia**). ^1^H-NMR δ 2.23 (s, 3H), 2.33 (s, 3H), 5.45 (s, 2H), 5.96 (s, 1H), 7.11 (d, *J* = 7.1 Hz, 2H), 7.19-7.66 (m, 5H), 8.45 (d, *J* = 7.9 Hz, 1H)

*3-Benzyl-2-methyl-10-(3-trifluoromethylbenzyl)dihydrobenzoDihydrobenzo[4,5]imidazo[1,2-a]pyrimidin-4-one*

(**5jg**). ^1^H-NMR δ 2.33 (s, 3H), 3.93 (s, 2H), 5.59 (s, 2H), 7.04-7.79 (m, 11H), 7.91 (s, 1H), 8.50 (d, *J* = 7.8 Hz, 1H).

*10-(2-Fluorobenzyl)-2-ethyldihydrobenzoDihydrobenzo[4,5]imidazo[1,2-a]pyrimidin-4-one* (**5kc**). ^1^H NMR δ 1.19 (t, *J* = 7.5 Hz, 3H), 2.56 (q, *J* = 7.5 Hz, 2H), 5.55 (s, 2H), 5.97 (s, 1H), 7.11 (m, 1H), 7.19-7.41 (m, 4H), 7.41-7.65 (m, 2H), 8.45 (dq, *J* = 0.7 Hz, *J* = 7.9 Hz, 1H); ^13^C-NMR δ 13.0, 31.1, 100.3, 110.4, 116.0, 116.4, 123.0, 125.3, 125.4, 126.0, 126.7, 130.7, 130.8, 131.4, 149.4, 160.0, 163.4, 170.0.

*2-tert-Butyl-10-(4-clorobenzyl)dihydrobenzoDihydrobenzo[4,5]imidazo[1,2-a]pyrimidin-4-one* (**5me**). ^1^H-NMR δ 1.27 (s, 9H), 5.48 (s, 2H), 6.03 (s, 1H), 7.13-7.59 (m, 6H), 7.66 (d, *J* = 8.1 Hz, 1H), 8.40 (d, *J* = 8.1 Hz, 1H).

*10-Butyl-2-trifluoromethyldihydrobenzoDihydrobenzo[4,5]imidazo[1,2-a]pyrimidin-4-one* (**5nj**). ^1^H-NMR δ 0.88 (t, *J* = 7.2 Hz, 3H), 1.31 (hexet, *J* = 7.5 Hz, 2H), 1.79 (pentet,*J* = 7.5 Hz, 2H), 4.32 (t, *J* = 7.2 Hz, 2H), 6.51 (s, 1H), 7.42 (t, *J* = 7.5 Hz, 1H), 7.60 (t, *J* = 7.6 Hz, 1H), 7.80 (d, *J* = 8.3 Hz, 1H), 8.50 (d, *J* = 8.0 Hz, 1H); ^13^C-NMR δ 13.9, 19.9, 30.2, 42.8, 100.1, 111.0, 116.6, 123.6, 124.6, 125.5, 127.6, 131.8, 149.6, 159.5, 160.2.
